# Child parent psychotherapy in the treatment of severe trauma in a 4-year-old child with co-occurring autism spectrum disorder

**DOI:** 10.1192/bjo.2021.543

**Published:** 2021-06-18

**Authors:** Clare Lamb, Barry O'Sullivan

**Affiliations:** London Infant & Family Team. South London & Maudsley NHS Foundation Trust / NSPCC

## Abstract

**Aims:**

This poster describes Child Parent Psychotherapy (CPP) in the treatment of severe trauma in a 4-year-old child with co-occurring Autism Spectrum Disorder (ASD).

**Background:**

The London Infant and Family Team (LIFT) implements the New Orleans Intervention Model. It targets the mental health needs of under 5 year olds, providing evidence based assessments and interventions for infants, their parents and foster carers within the framework of the Family Court in England. The majority of children seen by LIFT have suffered severe trauma. LIFT delivers a range of interventions including CPP - a relational treatment for young children who have experienced trauma.

CPP seeks to intervene in a number of ways: provides developmental guidance, demonstrates that the child's behaviour has meaning and can be linked to past traumas, enables the child to have space to play and talk about what has happened, helps to name and contain emotions - supporting emotional regulation, and helps the dyad to understand each other. The dyadic relationship is key to the intervention - helping to establish safety for the child and strengthen the caregiver-child relationship, enabling the child to make sense of past experiences and learn new ways to express feelings. Exploration of trauma takes place through a combination of play and interpretations made by the clinician, who supports and holds in mind the experiences and history of both child and carer. There is evidence that CPP helps young traumatised children to become less anxious, more secure in their attachment relationships and more able to cue their needs. There is less evidence of the efficacy of CPP in the context of young children with a co-occurring diagnosis of ASD.

**Method:**

The poster describes the assessment of a 4-year-old child of normal intelligence with a two year history of severe neglect, and physical and emotional abuse, who presented significant behavioural and emotional disturbance. Tools used to assess the child's behaviour, trauma symptoms and ASD are outlined. The process of CPP with the child and foster carer dyad is described. Outcome measures and symptom resolution are reported.

**Conclusion:**

Co-occurrence of ASD did not prevent this child accessing trauma therapy. He engaged in symbolic play, made use of CPP interpretations, and achieved significant improvement in his symptoms. The differential diagnoses of trauma symptoms and ASD presenting in young children are discussed, alongside the importance of understanding and treating trauma in this context.

